# Myeloid cell leukemia-1 expression in cancers of the oral cavity: a scoping review

**DOI:** 10.1186/s12935-022-02603-0

**Published:** 2022-05-06

**Authors:** Su-Jung Choi, Neeti Swarup, Ji-Ae Shin, Seong-Doo Hong, Sung-Dae Cho

**Affiliations:** Department of Oral Pathology, School of Dentistry and Dental Research Institute, Seoul National University, Seoul, 03080 Republic of Korea

**Keywords:** Mcl-1, Oral cavity, Cancer, Agents targeting Mcl-1

## Abstract

**Background:**

B cell lymphoma-2 (Bcl-2) family members play important roles in cell survival as well as cell death. The role of myeloid cell leukemia-1 (Mcl-1), an important member of the Bcl-2 family, is well established in hematopoietic malignancies. However, the association between Mcl-1 and oral cavity, cancers is not clearly defined.

**Methods:**

A scoping review was conducted until June 30, 2021, using four major databases, PubMed, Scopus, Web of Science, and Embase. Medical subject headings keywords for Mcl-1, along with its other identifiers, and head and neck cancers (only oral cavity tumors) were used to evaluate the expression, function, molecular association, and therapeutic approach of Mcl-1 in oral cavity cancers and precancers.

**Findings:**

Mcl-1 expression was associated with the progression of oral cavity cancers. The molecular mechanism and pathways of Mcl-1 in oral cavity cancers established via experimental results have been highlighted in this review. Moreover, the various synthetic and naturally derived therapeutic agents targeting Mcl-1 have been documented.

**Novelty/Improvement:**

Based on our present review, Mcl-1 appears to be an effective anticancer target that can be used in the therapeutic management of oral cancers.

## Background

### Cancerous lesions in the oral cavity

Oral cancers are a malignant tumors that occur in the mouth, and oropharynx and on the lips; oral cancers account for approximately 2% of all malignancies worldwide [1]. More than 90% of these cancers are squamous cell carcinomas (SCC) [2], and approximately 3 ~ 5% salivary gland tumors (SGTs) [3]. The potentially malignant lesions of the oral cavity (OPML) include conditions such as leukoplakia, erythroplakia, and submucous fibrosis [4]. Despite various advancements in therapeutic regimens, survival of patients with oral cancers has not significantly improved, and most chemotherapeutic or combination interventions have not been proven successful [5]. Thus, the identification of predictive molecules that preempt the malignant transformation to oral squamous cell carcinomas(OSCC) might prove to be useful in the development of effective therapies.

### Myeloid cell leukemia-1

Myeloid cell leukemia-1 (Mcl-1) was first identified in a myeloid leukemia cell line by Kozopas et al. in 1993 [6]. It is located at 1q.21, which is frequently amplified in cases of multiple myeloma [7]. Mcl-1 is involved in normal cell homeostasis and function. Under normal conditions, it protects the cells from apoptosis and plays an important role in cell survival. It also plays a significant role during embryogenesis. The deletion of this gene in murine embryonic stem cells resulted in peri-implantation embryonic lethality [8]. Mcl-1 also promotes the maintenance of normal mitochondrial morphology and energy production by exerting both anti-apoptotic and mitochondrial effects [9]. Just as anti-apoptotic Bcl-2 family members antagonize pro-apoptotic BH3-only proteins to inhibit the essential apoptosis effectors Bak/BAX [10], Mcl-1 exerts its anti-apoptotic function by sequestering the pro-apoptotic proteins Bak/BAX [11]. Mcl-1 is regulated via modifications at the transcriptional, post-transcriptional, translational, or post-translational levels, and the functional activity and stability of Mcl-1 is determined by its post-translational modifications [12–14]. Notably, alternative splicing can specifically affect Mcl-1 function by yielding a longer isoform, which is anti-apoptotic, or a shorter isoform, which is pro-apoptotic [13].

Mcl-1 overexpression has been associated with poor outcomes and therapeutic responses in hematologic malignancies [15] and breast [16, 17], lung [18], and gastric cancers [19]. Its overexpression in different cancers, particularly in leukemia, has resulted in an increased focus on the therapeutic targeting of this protein [20] leading to the development and identification of various synthetically produced, naturally occurring, or synthetically derived natural analogous compounds targeting Mcl-1 [21–24]. In addition to single compounds, combination therapies that target Mcl-1 reportedly show promising effects [24]. On the basis of the information currently available, we hypothesize that Mcl-1 can be a promising target for anticancer therapy.

The aim of the current review was to evaluate the expression, regulation, function, associated features, and potential therapeutic agents of Mcl-1 in oral cancers.

## Methods

A previously established method was used to conduct a scoping review by applying the Preferred Reporting Items for Systematic Reviews and Meta-Analyses for Scoping Reviews guidelines.

### Search strategy

**Fig. 1:**
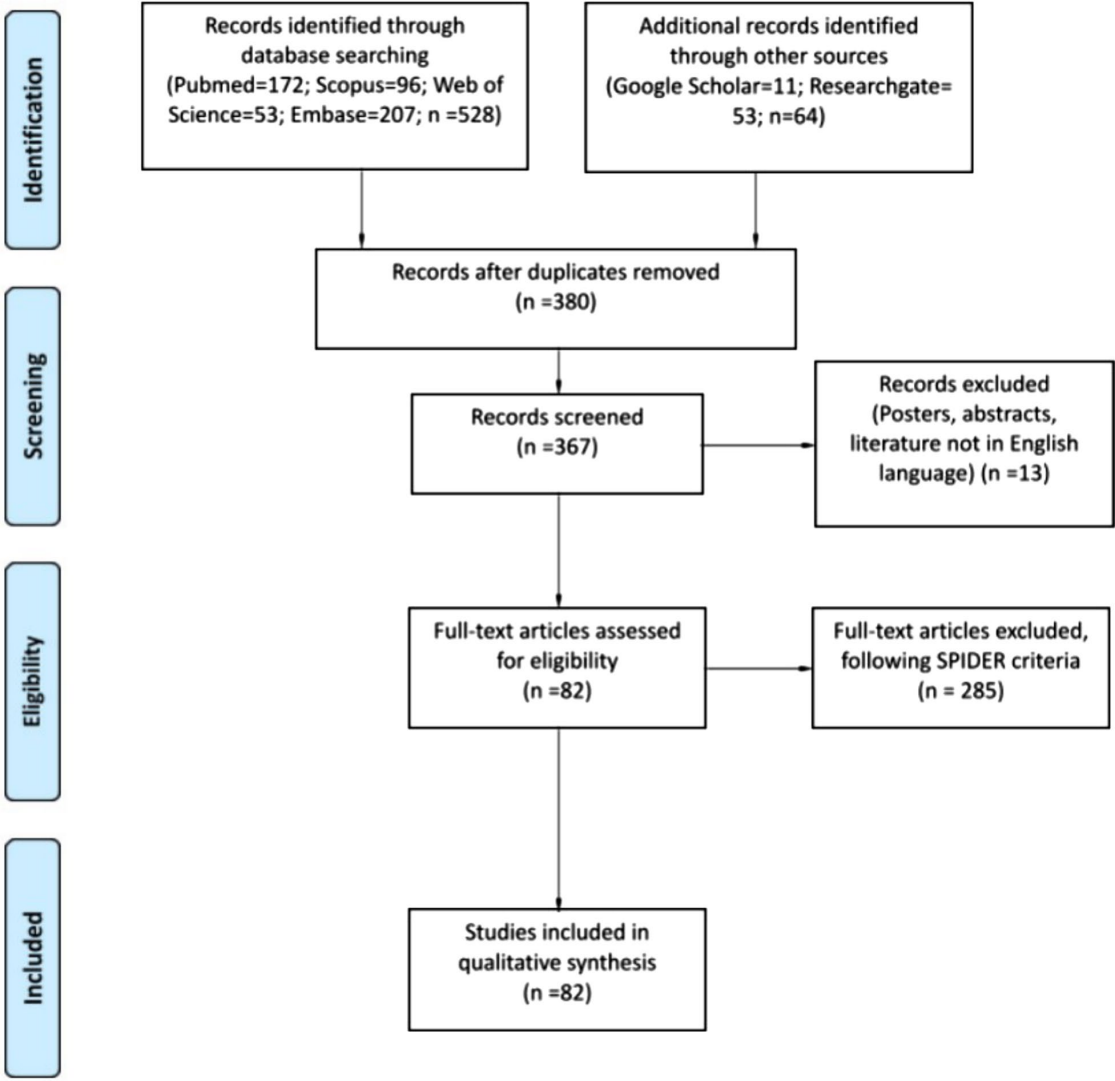
PRISMA flow chart of the scoping review. Summary of evidence search and selection

A literature search was conducted using the PubMed, Scopus, Embase, and Web of Science databases as well as a gray literature search using Research Gate and Google Scholar until June 30, 2021. Medical subject headings (MeSH) terms were used to explore Mcl-1 along with other aliases, such as oral cancer, SGT, precancerous lesion, head and neck SCCs; other tumors were not included for this review. Only the studies published in English were evaluated, and duplicated records, posters, and abstracts were excluded (Fig. 1).

### Eligibility criteria

The articles were reviewed by two authors of this study (SJC and NS) for eligibility and included after evaluation using the SPIDER criteria (Table 1).

**Table 1:** SPIDER inclusion criteria for literature evaluation

Sample	Excised human tissue samples; oral cancer cell lines; and *in vivo* models (using oral cancer cell lines or SGT cell lines)
Phenomenon of interest	Regulation, function, and therapeutic mechanisms (agents)
Design	Observational study, case study, focus group, and experimental studies
Evaluation	Characteristics and effects
Research type	Qualitative, quantitative, and mixed methods peer-reviewed studies and gray literature including third sector and government reports and briefings and educational theses

## Mcl-1 expression in clinical oral cancer samples

OPMLs have a high likelihood to progress to cancer, and the identification of oncogenic proteins that aid in the progression to oral cancer can be extremely helpful for better therapeutic planning. Several authors have verified that Mcl-1 is overexpressed in OPMLs. Ribeiro et al. [25] observed gains in Mcl-1 in two patients with leukoplakia and erythroleukoplakia. Similarly, Mallick et al. [26] reported the upregulation of Mcl-1 in malignant and premalignant tissues in vivo, interestingly indicating that the expression of Mcl-1 in homogeneous leukoplakia tended to be higher than in non-homogeneous leukoplakia. Our group also previously showed that Mcl-1 is overexpressed in oral lichen planus compared with the normal oral mucosa [27]. Sulkshane et al. reported that Mcl-1 was upregulated in OPMLs and demonstrated a positive correlation between Mcl-1 and USP9X in leukoplakia [28]. Moreover, Yu et al. found that an increase in the Bak/Mcl-1 ratio had favorable therapeutic outcomes after on photodynamic therapy for oral verrucous hyperplasia and leukoplakia [29]. These results indicate the essential role of Mcl-1 in the malignant transformation of OPMLs.

**Fig. 2:**
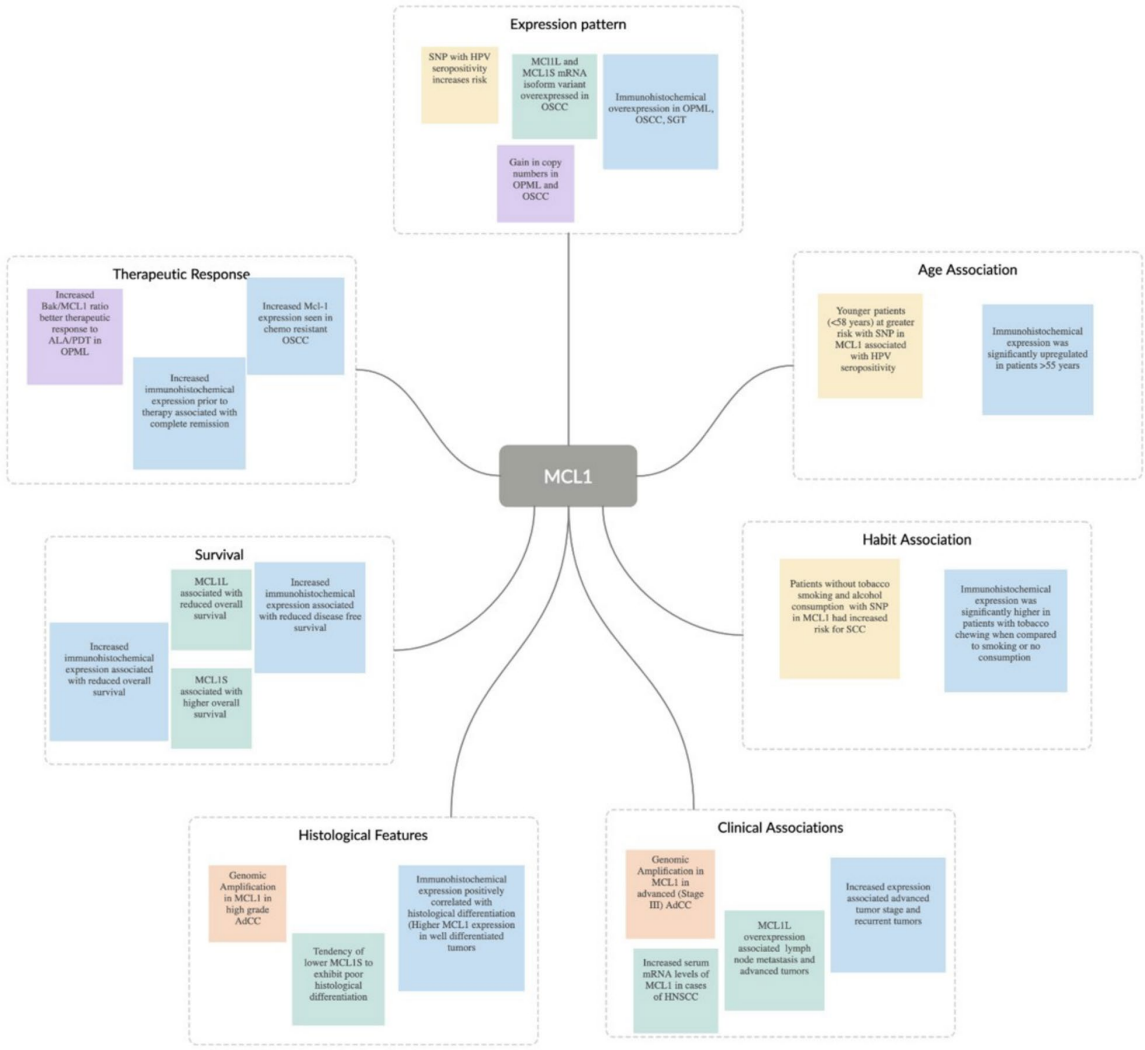
Scamper diagram showing Mcl-1 expression in oral cavity cancers and association with various clinicopathological features

Mcl-1 overexpression is well documented in various solid and hematological tumors, including oral cancer, and has been demonstrated as genetic amplifications [25] and in mRNA [26, 30, 31] and protein [26, 28, 32, 33] levels. According to a study by Nagata et al., strong Mcl-1 expression was observed in tongue SCC (SCCKN and SAS) cell lines compared with fibroblasts from normal lips [32]. The results of a study by Shin et al. [33] were valuable in terms of Mcl-1 expression through analysis of normal oral mucosa, human OSCC tissues (n = 14 and 25, respectively) and various OSCC cell lines (HSC2, HSC3, HSC4, HN22, OSC-20, Ca9.22, and SAS). In addition, Sulkshane et al. [28] confirmed the strong expression of Mcl-1 in other OSCC cell lines (AW8507, AW13516, and SCC029B). SGTs form a heterogeneous group of tumors that can be aggressive in nature; their gene expression patterns are similar to those of OPMLs and OSCC. The ubiquitous overexpression of Mcl-1 was reported in various types of malignant parotid gland tumors; the highest expression was observed in SCC of the parotid gland [34]. Although an isolated finding, Mcl-1 amplification was observed in high-grade stage III adenoid cystic carcinoma [35]. Determining the associations between Mcl-1 and the various categories and stages of oral cancer can enhance our understanding of its potential impact on the clinical progression of the disease. Studies on the associations between Mcl-1 overexpression and advanced tumor stages [28, 30] and lymph node metastasis [30] are limited. Mcl-1 overexpression has been reported more in recurrent tumors than in primary tumors [28]. In addition, increased Mcl-1 expression has been associated with reduced overall survival [28, 30], disease-free survival, and survival time [31, 36]. Various histopathological indicators have been used to predict the progression of OSCC. Interestingly, increased Mcl-1 expression was associated with well-differentiated tumors [26, 32]. Mcl-1 plays an important role in keratinocyte differentiation as it helps to maintain mitochondrial function [37]. These findings indicate a complex interaction, wherein histological function is maintained despite the poor clinicopathological stages. Taken together, the consistent findings of Mcl-1 overexpression in cancers indicates its association with carcinogenesis, and it is suggested that Mcl-1 has a significant impact on the development and progression of oral cancer. The associations between Mcl-1 and the different features of OPMLs, OSCC, and SGTs are summarized in Fig. 2; Table 2.

**Table 2:** Expression of Mcl-1 in oral cancers and precancerous lesions

Refs.	Subject sample type	Lesion type	Findings
[92]	Human HNSCC	Human HNSCC	MCL-1 positively correlated with Bak expression
[93]	Human HNSCC	HNSCC	Higher Mcl-1 associated with complete remission
[26]	Human OSCC, OPML	OPML, OSCC	Upregulated in OPML, OSCC; associated with well-differentiated OSCC
[32]	Human OSCC	OSCC	Upregulated in OSCC, associated with well-differentiated OSCC
[94]	Human OSCC	OSCC	MCL-1 associated with reduced disease-free survival
[95]	Human SGT	SGT	Upregulated in parotid tumors
[96]	Human HNSCC	Oropharyngeal cancer	SNP in MCL-1 in association with HPV16 associated with oropharyngeal cancer
[33]	Human OSCC	OSCC	MCL-1 upregulated in OSCC
[29]	Human OPML	OPML	Better therapeutic response with higher Bak/Mcl-1 ratio
[95]	Human serum with HNSCC	HNSCC	MCL-1 mRNA levels significantly upregulated in the serum of patients with HNSCC
[30]	Human OSCC	OSCC	MCL-1 L Upregulated in OSCC, associated with nodal metastasis, advanced tumor, reduced overall survivin
[35]	Human SGT	SGT	Amplified in one case, grade 3 AdCC
[27]	Human OPML	OPML	Upregulated in OPML
[25]	Human OPML, OSCC	OPML, OSCC	Amplified in OPML and OSCC
[38]	Human OSCC	OSCC	Increased expression of Mcl-1 in chemoresistant OSCC
[31]	Human OSCC	OSCC	Overexpression of Mcl-1 in OSCC when compared with adjacent normal tissues
[28]	Human OPML, OSCC	OPML, OSCC	Overexpression in OPML, OSCC. Associated with advanced tumors, recurrent tumors, and reduced overall survival

## Molecular associations of Mcl-1

Despite evidence on the role of Mcl-1 as an important molecular target in oral cancer, the molecular mechanisms involved in oral cancer have not been well documented compared with those in other cancers. Isolated reports on the regulation and interactions of Mcl-1 in oral cancer have been identified [32, 38, 39]. The activity of Mcl-1 in oral cancer is found to be regulated by paracrine signaling mechanisms, physical forces, or intracellular regulatory mechanisms [28, 39, 40].

**Fig. 3:**
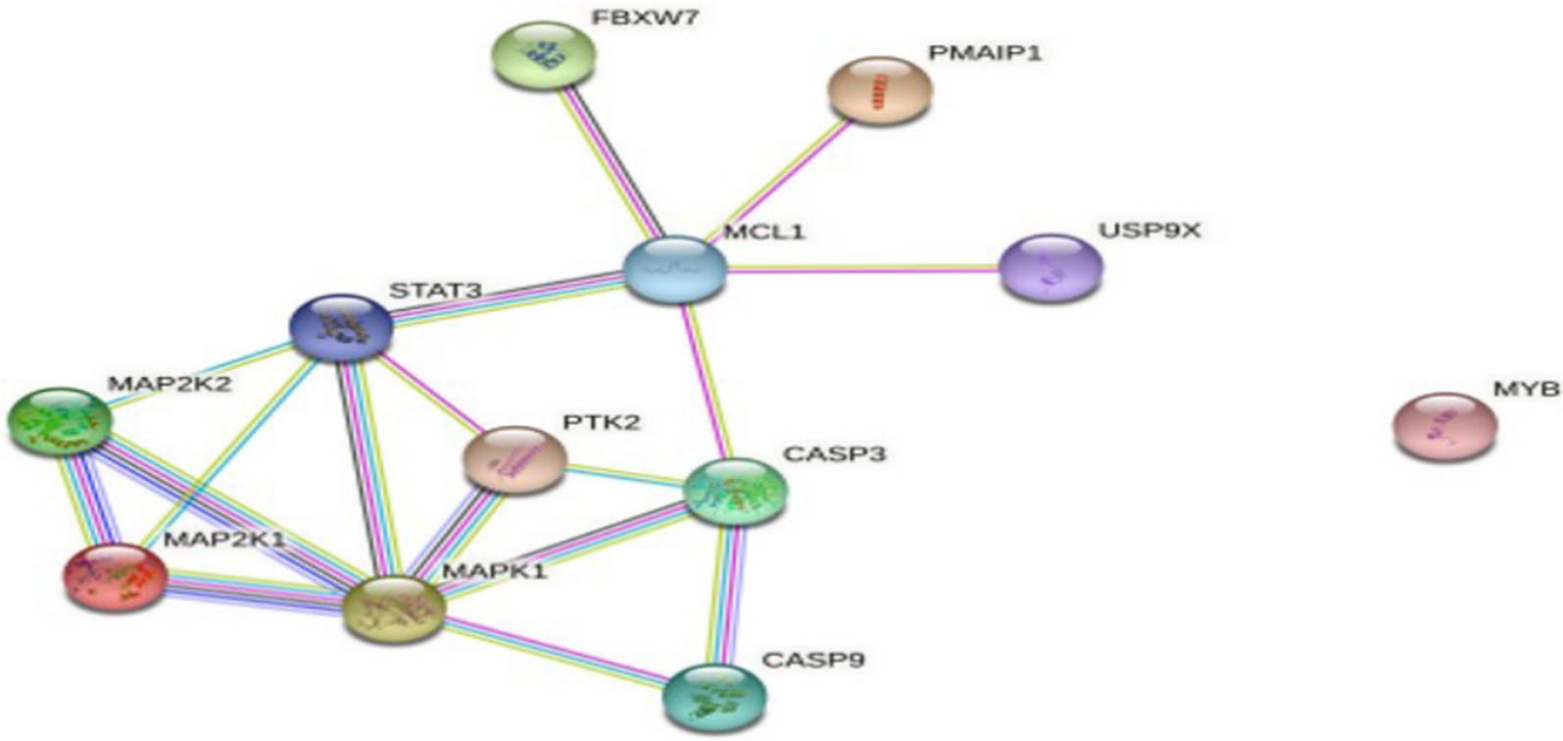
STRING protein–protein interaction (PPI) analyses. PPI network connectivity for proteins identified following the review. Nodes represent the proteins required for interaction. Edges represent the associations between the proteins. The STRING web resource (http://www.stringdb.org) was used in the prediction of the PPI (Protein–Protein Interaction) network whereby an interaction score of > 0.900 denoted a significant interactive relationship

The Mcl-1 mRNA expression was upregulated by STAT3 activation and stabilized by Akt-mediated GSK3β inactivation in chemotherapy-resistant OSCC [38]. The tumorigenesis regulating gene MYB is capable of upregulating Mcl-1 in adenoid cystic carcinoma cell lines [41]. FBW7 stabilizes Mcl-1 and promotes Mcl-1 addiction in oral cancer [42]. USP9X modulates the stability of Mcl-1 and prevents its degradation by deubiquitinating the protein [28]. Hyperosmotic stress has been shown to counteract Mcl-1 in head and neck SCC [39]. The upregulation of Noxa acts as a link between the osmotic pressure in the tumor environment and mitochondrial priming, thereby counteracting the anti-apoptotic properties of Mcl-1 in head and neck SCC. LncRNA FGD-AS1 inhibited the proliferation and migration/invasion of oral cancer, acting as a sponge for miR-153-3p and miR-153-3p to inhibit Mcl-1 expression [43]. Furthermore, the non-coding RNA HOXA10 AS was found to increase Mcl-1 mRNA levels [44]. Mcl-1 function can be also regulated through alternative splicing; a study demonstrated that Mcl-1 L transcripts were highly expressed compared with those of Mcl-1 S and Mcl-1ES in oral cells, thus indicating the predominance of the anti-apoptotic isoform [26, 30]. This variation in the isoform has a significant impact on Mcl-1 function and even on its clinical presentation [30, 45]. The effects of Mcl-1 on different oncogenic cascades have been evaluated in interference studies. Mcl-1 siRNA inhibited cell growth and induced apoptosis by inhibiting the FAK–MAPK pathway in OSCC [32]. Mithramycin inhibits Mcl-1 and RNAi regulates Bax to induce apoptosis in oral cancer cell lines [33]. These results suggest that Mcl-1 is affected and regulated by a variety of protein kinases, transcription factors, miRNA, etc. The molecular interactions and associations of Mcl-1 in oral cancers are summarized in Table 3, whereas and the protein–protein interactions (PPIs) between the identified biomarkers are presented in Fig. 3.

**Table 3:** Mechanistic associations of Mcl-1 in oral cancer

Factor/Phenomenon	Biological effects	Refs.
STAT3	Mcl-1 mRNA was upregulated by STAT3 activation. Mcl-1 protein was stabilized by Akt-mediated GSK3β inactivation It regulates chemoresistance in OSCC.	[38]
MYB	Mcl-1 expression was dependent on MYB expression	[41]
FBW7	FBW7 mutation stabilizes Mcl-1.	[42]
LncRNA FGD5-AS1	LncRNA FGD5-AS1 acted as an oncogene by regulating Mcl-1 via sponging miR-153-3p	[43]
Alternative splicing	Mcl-1 L transcripts overexpressed in oral cancer cell lines, and it was associated with poor prognostic indicators like advanced tumor size, lymph node metastasis, decreased survival, chemoresistance, and radioresistance	[30, 45]
HOXA10 antisense RNA (HOXA10-AS)	HOXA10-AS increased the stem cell property of OSCC stem cells via miR-29a/Mcl-1/PI3K/Akt signaling pathway	[44]
Noxa	Noxa binds to and sequesters Mcl-1, which releases Bak from Bak/Mcl-1 complex to be activated. Noxa overexpression enhanced the apoptotic effects of ABT-263	[39]
USP9X	Mcl-1 is primarily degraded by the ubiquitin–proteasome pathway in OSCC. USP9X interacts with Mcl-1 and stabilizes it to prevent its degradation	[28]
Mcl-1	p-FAK was decreased by treatment with Mcl-1 siRNA, resulting in decreases in phosphorylation of MEK1/2 and MAPK	[32]
Mcl-1	Inhibition of Mcl-1 leads to cellular apoptosis via caspase cascade via Caspase-3, 9	[33]

## Therapeutic strategy targeting Mcl-1

Various compounds that can result in apoptosis can reduce the expression level of Mcl-1 by inhibiting its translation or increasing its rate of degradation. These compounds have been found to have an effect on the levels of Mcl-1 when used alone or in combination with other agents. Therefore, the key factors that inhibit Mcl-1 can be used as potential treatment strategies in the treatment of oral cancer.

### Synthetic compounds

Several direct and indirect approaches to inhibit the activity of Mcl-1 have been used. Although small molecule inhibitors that directly target Mcl-1 by interrupting the PPIs have been developed, no drugs that can directly target this protein have been used in the treatment of oral cancer to date. Alternatively, some synthetic or natural compounds were found to target Mcl-1 indirectly as a part of their mechanism of action.

A Bcl-2 inhibitor, obatoclax, was found to induce apoptosis in head and neck SCC in an Mcl-1-dependent manner [46]. ABT-737 repressed cellular Mcl-1 by upregulating Noxa [47]. TW-37 was reported to sensitize cryptotanshinone-mediated apoptosis in OSCC cells by suppressing STAT3–Mcl-1 signaling [48]. Furthermore, the proteasome inhibitor MG132 induced the accumulation of Bik, which can activate Bak sequestered by Mcl-1, to sensitize the TRAIL-mediated apoptosis [49]. Several kinase inhibitors have been shown to downregulate Mcl-1 in oral cancer; e.g., the aurora-A kinase inhibitor, alisertib, degraded Mcl-1 in HPV E7-expressing head and neck SCC cells [50]. Similarly, the multikinase inhibitor sorafenib induced apoptosis in mucoepidermod carcinoma cells through the STAT3/Mcl-1/t-Bid signaling pathway [51]. EGFR inhibitors induced apoptosis in head and neck SCC by downregulating Mcl-1 expression [52, 53]. Mithramycin A reduced the expression of Mcl-1 in oral cancer cells, leading to an increase in Bax protein, followed by its translocation into the mitochondria and oligomerization [33]. An HDAC inhibitor, panobinostat, suppressed Sp1 and downregulated Mcl-1 levels [54]. An inhibitor of the splicing factor 3B1, meayamycin B, reportedly to inhibited SF3B, leading to a reduction in the anti-apoptotic Mcl-1 L isoform and the generation of the pro-apoptotic Mcl1-S by switching the splicing pattern of the Mcl-1 pre-mRNA [55]. YM155 inhibited Mcl-1 through lysosomal-dependent degradation to induce apoptosis in head and neck SCC cell lines [56]. Aspirin downregulated the Mcl-1 protein, followed by a significant reduction in ERK-1/2 and Akt phosphorylation and significant increase in IκB-α phosphorylation, thus resulting in the activation of NF-κB [57]. The immunosuppressant FTY720 downregulated Akt/NF-κB signaling through a Mcl-1-dependent mechanism [58]. Propofol induced apoptosis via a significant reduction in Mcl-1 and an increase in phospho-Mcl-1 (Ser 159) thereby indicating its effect on the stability of Mcl-1 protein [59]. Biochemical synthetic products such as glucosamine hydrochloride and the anti-malaria semi-synthetic dihydroartemisinin demonstrated a reduction in Mcl-1 in OSCC cell lines [60–63]. Several combination treatments affected the function of Mcl-1;e.g., a combination of fenretinide and ABT263 induced Mcl-1 degradation [64]. Co-treatment with C6 ceramide significantly augmented PKC412-induced lethality by downregulating Mcl-1 in head and neck cell lines and animal models [65]. These results suggest that synthetic compounds targeting Mcl-1 is a promising therapeutic strategy for the treatment of oral cavity cancers.

The combination of thioridazine and carboplatin induced apoptosis by downregulating c-FLIP and Mcl-1 [66], indicating that Mcl-1 can be used as a molecular target of combination therapy in oral cancer. Clinical studies on Mcl-1 inhibitors are under way, and anticancer effects have been identified in several cancers other than those of the oral cavity [31]. Venetoclax and others drugs are under clinical trials for the treatment of acute myeloid leukemia and other hematological malignancies [24]. Table 4 summarizes various synthetic agents used to target Mcl-1.

**Table 4:** Therapeutic strategy targeting Mcl-1 in oral cancer (synthetic)

Mechanism	Compound	Key findings	Study model	Refs.
Bcl-2 inhibitor	Obatoclax (GX15-070)	Obatoclax inhibited Mcl-1. The treatment led to the induction of HNSCC cell apoptosis in a Mcl-1-dependent manner. Its cytotoxicity increased following synergism with chloroquine (autophagy inducer)	UMSCC-1, Cal33	[46]
	ABT-737	ABT-737 alone or in combination with radiation led to repression of cellular Mcl-1 via Noxa upregulation. The combination between ABT 737 and radiation had a synergistic effect when compared with ABT 737 alone	SQ20B, SCC61, Cal27, Cal33	[47]
	Sabutoclax	OSCC cell survival was dependent on Mcl-1. Silencing Mcl-1 led to ABT 737-dependent cell death. Sabutoclax induced cancer-specific cell death in a Mcl-1-dependent manner. It also led to the induction of autophagy. Sabutoclax inhibited tumor growth in vivo. The effects were enhanced when used with celecoxib	H357, SCC-4, SCC-9, FaDu, In Vivo	[97]
	TW-37 (BH3 mimetic)	TW-37 induced apoptosis in OSCC cells by suppressing STAT3–Mcl-1 signaling. It also enhanced the effects of cryptotanshinone	HSC-3, Ca9.22, HSC-4	[48]
Proteasome inhibitor	MG132	MG132 sensitized HNSCC cells to apoptotic cell death mediated by DR5/DR4 ligand TRAIL or agonistic DR4 monoclonal antibody AY4. It inhibited the interaction of Bak with Mcl-1 and Bcl-xL via Bik	HN3, HN6	[49]
	Carfilzomib/IV ONX0912 (oprozomib)	Obatoclax inhibited Mcl-1. The treatment led to the induction of HNSCC cell apoptosis in Mcl-1-dependent manner. Its cytotoxicity increased following synergism with chloroquine (autophagy inducer)	UMSCC22A, 1483, UMSCC22B, UMSCC-1	[98]
Kinase inhibitor	Alisertib (MLN8237)	Aurora-A kinase inhibitor (Alisertib) led to degradation of Mcl-1 in HPV E7-expressing HNC cells. Cotreating with MG132 rescued Mcl-1 expression	SCC90, SCC104, SCC25	[50]
	Sorafenib	Sorafenib leads to proteasomal degradation of Mcl-1 and inhibition of translation. It can induce apoptosis through a STAT3/Mcl-1/t-Bid signaling pathway	MC3, YD15	[51]
	AZD-1775 (Wee-1 inhibitor)	AZD-1775 decreased the expression of the anti-apoptotic proteins, Mcl-1 and XIAP, by increasing the sensitivity of HPV + HNSCC cells to cisplatin	HPV16 + HNSCC cells, UMSCC47, HMS-001, HPV16- HNSCC cells, HN30(wtp53), HN31(mutp53), In vivo	[99]
EGFR inhibitor	Afatinib	Afatinib stimulates the PERK–eIF2α–ATF4 axis, which contributes to MCL-1 downregulation and subsequent apoptosis via suppressing Akt–mTOR signaling	FaDu, Detroit562, HN6, CAL-27	[53]
	SKLB188	SKLB188 induced caspase-dependent apoptosis by down-regulating Mcl-1 and survivin. It primarily inhibits the EGFR signaling	FaDu, PCI-13, In vivo	[52]
RNA synthesis inhibitor	Mithramycin A	Mithramycin A treatment led to the downregulation of Mcl-1. Mcl-1 inhibition led to an increase in pro-apoptotic protein Bax, resulting in the Bax translocation into mitochondria and its oligomerization	HN22, HSC4, *In vivo*	[33]
HDAC inhibitor	Panobinostat (LBH589)	Panobinostat treatment led to suppression of Sp1 protein, which led to Mcl-1, cyclin D1, and survivin. It also upregulated the expression levels of p27 and p21	HN22, HSC4	[54]
Splicing factor 3B1 inhibitor	Meayamycin B	Meayamycin B inhibited SF3B, which led to a reduction in anti-apoptotic Mcl-1 L isoform by modulating splicing of Mcl-1 mRNA. Stronger toxicity was seen in Mcl-1 abundant and HPV16 negative HNSCC cells	HPV + UD-SSC2, UM-SCC47, 93-VU-147T, UPCI: SCC90, HPV- PCI-13, PCI-15B, UM-SCC22B	[55]
Survivin inhibitor	Sepantronium bromide (YM155)	YM155 inhibited survivin, Sp1, and Mcl-1. Survivin and Mcl1 were inhibited via lysosomal-dependent degradation. Moreover, Sp1 inhibition also led to downregulation of Mcl-1	MC3, HN22	[56]
Antisense Oligonucleotides	Mcl-1 antisense oligonucleotides	Mcl-1 antisense oligonucleotides led to a significant reduction in Mcl-1 protein. Additionally, a synergistic cytotoxic effect was observed with cisplatin, 5-fluorouracil (5-FU), gemcitabine, paclitaxel, or cetuximab	SCC9	[100]
Acetylsalicylic acid (ASA)	Aspirin	Aspirin led to the downregulation of the Mcl-1 protein. Mcl-1 proteolysis was caspase dependent	YD8	[57]
	Aspirin + Sorafenib	Aspirin with sorafenib treatment had a synergistic impact on the induction of cell death. The combination treatment induces xCT inhibition, GSH depletion, and ROS accumulation. In addition, the combination of aspirin and sorafenib induced c-PARP and decreased p65, Mcl-1, and xCT protein expression	HN2-10, In vivo	[101]
Nonsteroidal anti-inflammatory drugs (NSAIDs)	Tolfenamic acid	Tolfenamic acid treatment led to inhibition of proliferation in cancer cells. It led to a reduction in Mcl-1 at both protein and mRNA levels via Sp1	YD15	[79]
Immunosuppressant	FTY720	FTY720 induced downregulation of Akt/NF-κB pathway, ROS generation, Mcl-1 degradation, and autophagy-dependent apoptosis in OSCC cells.	SCC2095	[58]
Chemotherapy medication	Vincristine	Vincristine treatment induces HMGB1 release leading to autophagy, which protects oral cancer cells. HMGB1 increases Mcl-1 expression via RAGE signaling and protects the cells from vincristine-induced apoptosis	SCC9, OECM-1	[102]
	Fenretinide + ABT-263	Fenretinide treatment along with ABT-263 significantly induced apoptosis. MCL-1 and BCL-xL are the primary targets of apoptosis induced by ABT-263 in combination with Ad-Noxa or fenretinide	HN8, HN12, HN30, UMSCC1, UMSCC47, UMSCC104	[64]
	C6 ceramide + PKC412	C6 ceramide co-treatment significantly augmented PKC412-induced lethality in HNSCC cells. Together they synergistically decreased Akt–mTOR activation. C6 ceramide sensitized the cells to PKC412 via down-regulating Mcl-1.	SQ20B, SCC-9	[65]
Antipsychotic drug	Thioridazine + curcumin	Thioridazine and curcumin combined treatment induced apoptosis through down-regulating c-FLIP and Mcl-1 expression at the post-translational levels via NOX4-mediated upregulation of proteasome activity	AMC-HN4	[66]
	Thioridazine + carboplatin	Thioridazine plus carboplatin induces apoptosis in human head and neck cancer cells. The augmentation of proteasome activity by mitochondrial ROS-mediated PSMA5 expression induced the downregulation of c-FLIP and Mcl-1 expression in thioridazine plus carboplatin-treated cells	AMC-HN4	[103]
Anesthesia	Propofol	Propofol treatment led to the induction of apoptosis via inducing GAS5 via FoxO1. GAS5 down-regulates mir-1297 which suppresses GSK3β. This led to a significant reduction of Mcl-1.	UM-SCC6, SCC090	[59]
Biochemical synthetic product	Glucosamine hydrochloride (GS-HCl)	GS-HCl significantly reduced proliferation and induced apoptosis. It transcriptionally repressed Mcl-1 and HIF-1α in a concentration-dependent manner. Additionally, it also led to the mitochondrial-dependent activation of caspases and triggered ER stress	YD-8	[62]
	Fucoidan	Fucoidan treatment significantly induces apoptotic cell death by inactivating the ERK1/2 pathway thereby regulating the Mcl-1 protein	MC3	[63]
	Naphtho[1,2-b] furan-4,5-dione	NFD treatment led reduction in cancer cell growth. It led to the phosphorylation of EGFR. This led to a reduction in phosphorylation of PI3K and Akt. Dysregulation in PI3K and Akt led to a reduction in Mcl-1. Additionally, it led to downregulation of NF-κB and phosphorylation of IκKβ.	Ca9-22, SAS, CAL27	[60]
	Triptolide	Triptolide treatment led to the inhibition of Mcl-1 mRNA levels. It synergistically enhanced chemotherapy-induced cell death in chemoresistant OSCC	H357, SCC4	[38]
Anti-malaria &semi-synthetic product	Dihydroartemisinin	Dihydroartemisinin treatment induced antitumor effects. The antitumor effects were due to the inhibition of STAT3 via Jak2 kinase. The downstream targets of STAT3 including Mcl-1, Bcl-xL, Cyclin-D1, and VEGF were down-regulated	FaDu, Cal-27, In Vivo	[61]

### Natural compounds

Many natural compounds are known to affect STAT3, which is known as one of the major upstream molecules of Mcl-1 in oral cancers [67]. Epigallocatechin gallate abrogated the interleukin-6-induced phosphorylation of STAT3 and downregulated its target gene products [68]. Licochalcone C inhibited the JAK2/STAT3 pathway, and downregulated Bcl-2 and Mcl-1 [69]. Nitidine chloride decreased the Mcl-1 protein by inhibiting the STAT3 pathway [70]. Additionally, bitter melon extract inhibited the c-Met signaling pathway and reduced the downstream signaling molecules such as phospho-STAT3 (Tyr705) and Mcl-1 [71]. These findings suggest that the STAT3/Mcl-1 signaling axis is a promising molecular mechanism that can be used in the treatment of oral cancers.

Various phytochemicals may mimic the effects of BH-3 proteins. Guggulsterone phytosterol targets 14-3-3 zeta to initiate apoptosis through the intrinsic mitochondrial pathway by the dephosphorylation of p-Bad and suppression of the expression level of Mcl-1 in OSCC cells [72]. Furano-1,2-naphthoquinone upregulated Bax and Bad and downregulated Mcl-1 in Ca9.22 cells [73]. *Convallaria keiskei* reduced the expression level of Mcl-1, leading to a truncated Bid-induced mitochondrial apoptosis in salivary gland cancer cell lines [74]. Lycorine hydrochloride induced the mitochondria-mediated apoptosis pathway through the downregulation of Mcl-1 [75]. Treatment with *Juniperus squamata* induced a mitotic catastrophe, leading to apoptosis via Mcl-1 reduction in OSCC cell lines [76].

Extracts from various plants were found to target Sp1, which combines with a specific DNA sequence and is overexpressed in many cancers [77]. Sp1, a transcription factor that binds to the Mcl-1 promoter region [78], has already been tested and found to play important physiological roles, such as in apoptosis, by targeting Mcl-1 in cancer [54, 79]. Honokiol inhibited Sp1 and reduced Mcl-1 and survivin leading to the induction of apoptosis in OSCC cells [80]. Manumycin A inhibited Mcl-1 by downregulating Sp1 [81]. *Sanguisorba officinalis* [82] and *C*. *officinale Makino*, *C*. *bursapastoris* [83], and *Dianthus chinensis* and *Acalypha australis* [84] were found to reduce Mcl-1 via Sp1 and induce apoptosis in oral cancer cell lines.

ROS production results in a reduction in the mitochondrial transmembrane potential which leads to mitochondria-dependent apoptosis in human cancer cells [85]. ROS has been implicated in the activation of various cellular signaling pathways and transcription factors [86]. Phenethyl isothiocyanate induced G2/M cell cycle arrest and apoptosis by inducing ROS production and reducing Mcl-1 expression [87]. Benzyl isothiocyanate led to a reduction in Mcl-1 followed by the development of mitochondria-mediated apoptosis in oral cancer [88]. Cardiac glycosides induced apoptosis by lowering Mcl-1 levels in OSCC cell lines [89]. Wogonin was noted to selectively kill cisplatin-resistant head and neck SCC cells by targeting Nrf2, which was then accompanied by the downregulation of Mcl-1 [90]. Cyclocommunol downregulated the phosphorylation/expression of Akt/mTOR and Mcl-1 leading to the generation of ROS [91]. Taken together, the most commonly observed mechanism of action of these natural compounds in the regulation of Mcl-1 was through the inhibition of STAT3 or Sp1. Table 5 presents an overview of the effects of the natural compounds on Mcl-1.

**Table 5:** Therapeutic strategy targeting Mcl-1 in oral cancer (natural agents)

Active compound	Plant/Organism	Key findings		Model		Refs.
(−)-Epigallocatechin gallate (EGCG) polyphenol	Polyphenol	EGCG treatment led to an increase in Fas/CD95 death receptors, leading to caspase-8 activation. Reduction in levels of phosphorylated STAT3 (Tyr705 and Ser727) via interleukin-6 (IL-6) induced reduction in phosphorylated Jak1/2, following EGCG treatment. Reduction in STAT3 was associated with a reduction in Mcl-1		SAS, Cal-27, Ca9-22		[68]
Fisetin	Flavonoid	Fisetin-induced apoptotic cell death via induction of ROS, ER stress, and by disrupting the mitochondria membrane potential, which caused cytochrome c, AIF, and ENDO G release from mitochondria into the cytosol. It also led to a reduction in the expression of Mcl-1 and other apoptotic markers		HSC3		[104]
		Fisetin suppressed cellular growth, via modulating the SESN2/mTOR/Mcl-1 signaling axis		MC3, Ca9.22, HN22		[105]
Honokiol (HK)	*Magnolia officinalis* or *grandiflora*	Honokiol treatment led to a reduction in Sp1 expression. It was also associated with a significant reduction in Mcl-1 and survivin and upregulation in p21 and p27 resulting in caspase-dependent apoptosis		HN-22, HSC-4		[80]
Licochalcone A	The root of *Glycyrrhiza inflata*	Licochalcone A treatment led to a reduction in OSCC cell growth via downregulation of Sp1 expression and subsequent regulation of Sp1 downstream proteins such as p27, p21, cyclin D1, Mcl-1, and survivin		HSC4, HN22		[106]
Licochalcone B	*Retro chalcone* family (root of *Glycyrrhiza glabra or Glycyrrhiza inflata*)	Licochalcone B treatment induced apoptosis in OSCC cells by up-regulating the death receptor and modulating the Bcl-2 family members (downregulation of Mcl-1)		HN22, HSC4		[107]
Licochalcone C	*Retro chalcone* family (roots of *Ccardihinese licorice*)	Licochalcone C treatment modulated the Jak2 activity by physically binding to it. The binding led to a reduction in phosphorylation of Jak2. This led to a reduction in phosphorylated STAT3 levels and subsequently its downstream targets such as Bcl-2, Mcl‐1, and survivin		HN22, HSC4		[69]
Dehydroandrographolide (DA) diterpene	*Andrographis paniculata* (Burm.f.) Nees (family, *Acanthaceae*)	Dehydroandrographolide treatment induces autophagy, which is mediated via Beclin-1 by inhibiting Bcl-2, Bcl-xL, and Mcl-1. Additionally, it also led to inhibition of Akt, p38 phosphorylation, and enhanced JNK1/2 signaling pathways		SAS, OECM-1		[108]
Oridonin	*Rabdosia rubescens*	Oridonin treatment induced apoptosis via downregulation of Mcl-1. Mcl-1 downregulation led to the subsequent loss of MOMP and t-Bid		MC3, YD15		[109]
Evodiamine quinolone alkaloid	*Evodia fructus*	Evodiamine induced apoptosis by down-regulating Mcl-1 mRNA and protein. The downregulation in Mcl-1 was due to a reduction in Akt phosphorylation		MC3, HSC4		[110]
Cryptotanshinone (CT), tanshinones	Root of *Salvia miltiorrhiza*	Cryptotanshinone treatment modulated STAT3 activity and caused cell death. Reduction in STAT3 phosphorylation led to a reduction in survivin at the transcriptional level and reduced the activity of Mcl-1 via proteasomal degradation		MC3, YD15		[111]
Nitidine chloride (NC) quaternary ammonium alkaloid	*Zanthoxylum nitidium*	NC treatment led to a reduction in Mcl-1 via lysosomal-dependent degradation. The reduction in Mcl-1 following NC treatment was greater than that caused by STAT3 inhibitors		HSC3, HSC4, In vivo		[70]
Reserpine indole alkaloid	*Rauwolfia serpentina*	Reserpine treatment promoted apoptosis in DMBA-induced tumors in mice, like reduction in Mcl-1. Additionally, it inhibited TGF-β signaling, DNA repair protein expression, and proliferative and invasive proteins		HEC59 (Chemical induced carcinogenesis in an animal model)		[112]
Phenethyl isothiocyanate isothiocyanate	*Cruciferous* vegetable	Phenethyl isothiocyanate treatment led to cellular apoptosis and inhibited proliferation. The reduction in Mcl-1 levels was induced via GSH redox stress trigger. ROS (reduction in ΔΨm)		OC2, SCC4, SCC25		[87]
Benzyl isothiocyanate (BITC)	Plants of the mustard family	Benzyl isothiocyanate treatment led to cellular apoptosis and inhibited proliferation. It was associated with reduced mitochondrial potential ROS (reduction in ΔΨm). The reduction in Mcl-1 levels was induced via GSH redox stress trigger		OC2		[88]
Divaricoside cardiac glycosides	*Strophanthus divaricatus*	Divaricoside treatment suppressed the viability of OSCC cells. In addition to ROS generation, DIV induces autophagy and modulates the antitumor activity by lowering Mcl-1 levels in OSCC cells		SCC2095		[89]
α-l-Diginoside cardiac glycosides	*Strophanthus divaricatus* (*Apocynaceae*)	α-l-Diginoside treatment inhibited cellular proliferation. It inhibited Mcl-1 via proteasomal degradation. Additionally, it modulates Jak/Stat signaling		SCC2095, SCC4		[113]
Manumycin A (Manu A) natural antibiotic	*Streptomyces parvulus*	Manumycin A treatment resulted in Sp1 mediated apoptosis. It reduced Sp1 protein levels, thereby modulating its downstream targets like increasing p27 and p21, and decreasing Mcl-1 and survivin		HN22, HSC4		[81]
Guggulsterone phytosteroid	*Commiphora mukkul*	Guggulsterone treatment led to effective cytotoxic activity by inducing apoptosis in chemoresistant cancer cells. It targets 14-3-3 zeta to initiate apoptosis through the intrinsic mitochondrial pathway by releasing Bad from its inhibitory action. Additionally, it suppressed the expression of anti-apoptotic proteins xIAP, Mcl-1, c-myc, and survivin in SCC4 cells		SCC4, HSC2		[72]
Wogonin flavonoid	*Scutellaria baicalensis* Georgi	Wogonin treatment had significant cytotoxic effects. It targets the Nrf2-ARE pathway associated with chemotherapeutic resistance along with Mcl-1. Additionally, it induces intracellular ROS accumulation and GSH depletion. This leads to the potentiation of cisplatin cytotoxicity		AMC-HN4-cisR, HN9-cisR		[90]
Cyclocommunol (CYC) prenylflavonoid	*Artocarpus altilis*	CYC treatment caused pro-apoptotic effects via down-regulating the phosphorylation/expression of Akt/mTOR and Mcl-1		SCC2095, Ca922		[91]
Furano-1,2-naphthoquinone (FNQ) iNOS inhibitor	*Avicennia marina*	FNQ treatment led to cellular apoptosis via upregulation of Bax, Bad, and downregulation of Bcl-2, Bcl-xL, Mcl-1, and XIAP, resulting in cytochrome C release and sequential activation of caspase-9 and caspase-3. Additionally, it inactivated Src and PI3K/Akt-mediated cell signaling, which led to cell cycle arrest		Ca9-22, SAS, CAL27		[73]
Cardiotoxin III	*Naja naja atra*	Cardiotoxin III treatment abrogated the activation of EGFR and downstream events including phosphorylation of STAT3, STAT5, Akt, and ERK1/2. Moreover, it upregulated Bax expression and downregulated Bcl-2, Bcl-xL, and Mcl-1 expression		Ca9-22		[114]
Water extract of *Sanguisorba officinalis*	*Sanguisorba officinalis*	HESO treatment led to reduced cell growth and induced apoptosis in HSCC4 and HN22. In the HSC4 cell line, HESO reduced Mcl-1, which led to the activation and oligomerization of Bak, whereas in the HN22 cell line, HESO decreased Sp1 and its downstream target, survivin.		HSC4, HN22		[82]
Methanol extract of *C*. *officinale Makino* and *C*. *bursa*‑*pastoris*	*C. officinale Makino* *C. bursapastoris*	MECO and MECB treatment led to a reduction in cellular viability. It led to downregulation in Sp1 levels. Mcl-1 was down-regulated as a downstream target for Sp1		HSC2		[83]
Methanol extract of *Dianthus chinensis* and *Acalypha australis*	*Dianthus chinensis* and *Acalypha australis*	MEDC and MEAL treatment led to a reduction in cellular viability. It led to downregulation in Sp1 levels. Mcl-1 was down-regulated as a downstream target for Sp1		YD15, SCC15		[84]
Bitter melon	*Momordica charantia*	BME treatment led to inhibition in cellular proliferation. The treatment led to the inhibition and downregulation of c-met and its downstream targets, such as phospho-STAT3 (Tyr705) and Mcl-1 (long anti-apoptotic form). Additionally, a reduction in c-myc was also observed.		Cal27 (tongue), JHU-22 (Larynx), JHU-29(tongue)		[71]
Methanol extract of *Convallaria keiskei*	*Convallaria keiskei*	MECK treatment led to increased cell death. It induced Mcl-1 downregulation in a translation-dependent manner. Mcl-1 downregulation resulted in truncated Bid-induced mitochondrial apoptosis and downregulation in ERK1/2 phosphorylation		MC3, HN22		[74]
Lycorine hydrochloride	*Lycoris radiate*	Lycorine hydrochloride treatment inhibited the proliferation of OSCC cells. It induces the mitochondrial pathway and is involved in ROS-mediated apoptosis. It upregulated the expression levels of the pro-apoptotic members, Bax and Bim, but down-regulated the expression of the anti-apoptotic protein, Mcl-1, in a dose-dependent manner		HSC3		[75]
Ethanolic extract of *Juniperus squamata*	*Juniperus squamata*	EEJS treatment had cytotoxic effects on OSCC cells. It induced mitotic catastrophe, which led to apoptosis, via Mcl-1 reduction		HSC3, HSC4		[76]

## Conclusions

In this paper, we attempted to review the expression, function, molecular mechanism and pathway, and therapeutic approach of Mcl-1 in oral cavity cancers. Mcl-1 is frequently amplified and upregulated in cancerous lesions of oral cavity and affects the clinical progression and survival of patients with oral cancer. Various transcription factors and protein kinases affect Mcl-1 activity, which further facilitates cancer progression. These findings indicate its significant role in oral carcinogenesis. This review also successfully summarized the agents, both synthetic and natural, that have an inhibitory effect on Mcl-1 in oral cancer. To the best of our knowledge, this review is the first specific summary suggesting that Mcl-1 is a promising molecular target for the treatment of oral cancer. Although the development of direct Mcl-1 inhibitors remains challenging, this review will help researchers and clinicians to identify the avenues that can be investigated to provide better disease prediction and therapeutic planning of oral cancers expressing Mcl-1 in the future.

## Data Availability

Not applicable.

## Abbreviations

MeSH: Medical subject headings
PPI: Protein–protein interaction
SCC: Squamous cell carcinomas
SGT: Salivary gland tumors
TNBC: Triple negative breast cancer
